# Lower-Limb Lymphedema after Sentinel Lymph Node Biopsy in Cervical Cancer Patients

**DOI:** 10.3390/cancers13102360

**Published:** 2021-05-13

**Authors:** David Cibula, Martina Borčinová, Simone Marnitz, Jiří Jarkovský, Jaroslav Klát, Radovan Pilka, Aureli Torné, Ignacio Zapardiel, Almerinda Petiz, Laura Lay, Borek Sehnal, Jordi Ponce, Michal Felsinger, Octavio Arencibia-Sánchez, Peter Kaščák, Kamil Zalewski, Jiri Presl, Alicia Palop-Moscardó, Solveig Tingulstad, Ignace Vergote, Mikuláš Redecha, Filip Frühauf, Christhardt Köhler, Roman Kocián

**Affiliations:** 1 Gynecologic Oncology Center, Department of Obstetrics and Gynecology, First Faculty of Medicine, Charles University and General University Hospital, 12000 Prague, Czech Republic; martina.borcinova@vfn.cz (M.B.); filip.fruehauf@vfn.cz (F.F.); roman.kocian@vfn.cz (R.K.); 2 Department of Special Operative and Oncologic Gynaecology, Asklepios-Clinic Hamburg, 22763 Hamburg, Germany; simone.marnitz-schulze@uk-koeln.de (S.M.); ch.koehler@asklepios.com (C.K.); 3 Data Analysis Department, Institute of Biostatistics and Analyses, Faculty of Medicine, Masaryk University, 62500 Brno, Czech Republic; jarkovsky@iba.muni.cz; 4 Department of Obstetrics and Gynecology, University Hospital Ostrava, 70800 Ostrava Poruba, Czech Republic; jaroslav.klat@fno.cz; 5 Department of Obstetrics and Gynecology, Faculty of Medicine and Dentistry, Palacky University, University Hospital Olomouc, 77900 Olomouc, Czech Republic; radovan.pilka@fnol.cz; 6 Unit of Gynecological Oncology, Institute Clinic of Gynaecology, Obstetrics, and Neonatology, Hospital Clinic-Institut d’Investigacions Biomediques August Pi i Sunyer (IDIBAPS), University of Barcelona, 08036 Barcelona, Spain; atorne@clinic.cat; 7 Gynecologic Oncology Unit, La Paz University Hospital, 28046 Madrid, Spain; ignacio.zapardiel@uam.es; 8 Serviço de Ginecologia, Instituto Portugues de Oncologia do Porto, 1099-023 Porto, Portugal; almerindapetiz@ipoporto.min-saude.pt; 9 Department of Gynaecology, Institute of Oncology Angel H Roffo University of Bueno s Aires, Buenos Aires C1417 DTB, Argentina; consultas@dralauralay.com; 10 Department of Obstetrics and Gynecology, First Faculty of Medicine, University Hospital Bulovka, Charles University, 18081 Prague, Czech Republic; borek.sehnal@bulovka.cz; 11 Department of Gynecology, Biomedical Research Institute of Bellvitge (IDIBELL), University Hospital of Bellvitge, University of Barcelona, 08908 Barcelona, Spain; jponce@bellvitgehospital.cat; 12 Department of Gynecology and Obstetrics, Faculty of Medicine, Masaryk University, 60177 Brno, Czech Republic; felsinger.michal@fnbrno.cz; 13 Departments of Gynecologic Oncology, University Hospital of the Canary Islands, 35016 Las Palmas de Gran Canaria, Spain; oaresan@gobiernodecanarias.org; 14 Department of Obstetrics and Gynecology, Faculty Hospital Trencin, 911 71 Trencin, Slovakia; Peter.kascak@fntn.sk; 15 Department of Gynecologic Oncology, Holycross Cancer Center, 25-734 Kielce, Poland; SCOinfo@onkol.kielce.pl; 16 Department of Obstetrics and Gynecology, Faculty of Medicine Pilsen, University Hospital in Pilsen and Charles University, 30460 Pilsen, Czech Republic; preslj@fnplzen.cz; 17 Gynecology Department, Instituto Valenciano de Oncologia (IVO), 46009 Valencia, Spain; apalop@fivo.org; 18 Department of Obstetrics and Gynecology, Trondheim University Hospital, 7030 Trondheim, Norway; Solveig.Tingulstad@stolav.no; 19 Department of Gynecology and Obstetrics, Leuven Cancer Institute, University Hospital Leuven, 3000 Leuven, Belgium; ignace.vergote@uzleuven.be; 20 Department of Gynaecology and Obstetrics, University Hospital, Comenius University, 814 99 Bratislava, Slovakia; redecha2@uniba.sk

**Keywords:** cervical cancer, sentinel lymph node biopsy, lower limb lymphedema, pelvic lymphadenectomy

## Abstract

**Simple Summary:**

Lower-limb lymphedema (LLL) is a well-recognized adverse outcome of the surgical management of cervical cancer. Recently, sentinel lymph node (SLN) biopsy has emerged as an alternative procedure to systematic pelvic lymphadenectomy (PLND) aiming to decrease the risk of complications, especially LLL development. Our study represents the first prospective analysis of LLL incidence in cervical cancer patients after a uterine procedure with SLN biopsy, without systematic PLND. In an international multicenter trial SENTIX, the group of 150 patients was prospectively evaluated using both objective and subjective LLL assessments in 6 months intervals for 2 years. Contrary to the expectations, our results showed that SLN biopsy does not eliminate the risk of LLL development which occurred in a mild or moderate stage in about 26% of patients with a median interval to the onset of 9 months.

**Abstract:**

Background: To prospectively assess LLL incidence among cervical cancer patients treated by uterine surgery complemented by SLN biopsy, without PLND. Methods: A prospective study in 150 patients with stage IA1–IB2 cervical cancer treated by uterine surgery with bilateral SLN biopsy. Objective LLL assessments, based on limb volume increase (LVI) between pre- and postoperative measurements, and subjective patient-perceived swelling were conducted in six-month periods over 24-months post-surgery. Results: The cumulative incidence of LLL at 24 months was 17.3% for mild LLL (LVI 10–19%), 9.2% for moderate LLL (LVI 20–39%), while only one patient (0.7%) developed severe LLL (LVI > 40%). The median interval to LLL onset was nine months. Transient edema resolving without intervention within six months was reported in an additional 22% of patients. Subjective LLL was reported by 10.7% of patients, though only a weak and partial correlation between subjective-report and objective-LVI was found. No risk factor directly related to LLL development was identified. Conclusions: The replacement of standard PLND by bilateral SLN biopsy in the surgical treatment of cervical cancer does not eliminate the risk of mild to moderate LLL, which develops irrespective of the number of SLN removed.

## 1. Introduction

Lower limb lymphedema (LLL) ranks amongst the most serious types of enduring postoperative morbidity following surgical lymph node staging in gynecological cancer patients [1]. It represents a manifestation of lymphatic system insufficiency and deranged lymph transport [2] and is characterized by swelling of one or both lower limbs caused by excess accumulation of water, plasma proteins, extravascular blood cells, and parenchymal/stromal cell products [3]. LLL significantly decreases the quality of life of gynecological cancer survivors, negatively affecting daily life activities as well as their social and sexual life [4]. Since the worldwide median age of cervical cancer diagnosis is mid-to-late 40s, with 25% diagnosed under the age of 40, minimizing the long-term risk of LLL in this relatively young population with an excellent prognosis in early stages is of particular importance [5,6].

The true incidence of LLL after surgical treatment of cervical cancer is unclear, previously reported in the range of 0–62% [7,8,9,10,11,12,13,14,15]. The incidence varies mainly according to the extent of the surgery and administration of adjuvant treatment [16,17,18,19]. Namely, the number of removed lymph nodes has been shown to be directly related to the LLL development; however, the critical number of lymph nodes varied from 10 to 31 [9,15,20,21,22,23].

The main motive for introducing sentinel lymph node (SLN) biopsy as a replacement of standard systematic pelvic lymphadenectomy (PLND) is to decrease postoperative morbidity, specifically the development of LLL. Since the average number of SLN among cervical cancer patients is between 2–4 and removal of SLN does not usually require a complete dissection of pelvic spaces and interruption of all main lymphatic trunks, substantial reduction of LLL is expected after SLN biopsy without systematic PLND [24,25,26]. Implementation of SLN biopsy was already shown to be an effective strategy in reducing the incidence of LLL after treatment of endometrial or vulvar cancer [27,28,29,30]. On the other hand, only a few studies reported the incidence of LLL in cervical cancer patients after SLN biopsy without simultaneous PLND, mostly being retrospective and with substantial flaws in the methodology of LLL assessment [25,26,31].

The aim of the present study was to perform a pre-planned analysis of the incidence of LLL in a cohort of 150 patients who reached at least 24 months follow-up in a prospective international multicenter cohort study, SENTIX.

## 2. Materials and Methods

### 2.1. Study Design and Participants

In this pre-planned interim analysis, data were analyzed from 150 patients who were treated per protocol in the SENTIX trial and had two years of follow-up data available. Patients were enrolled in the study between 05/2016 and 11/2017.

**Figure 1:**
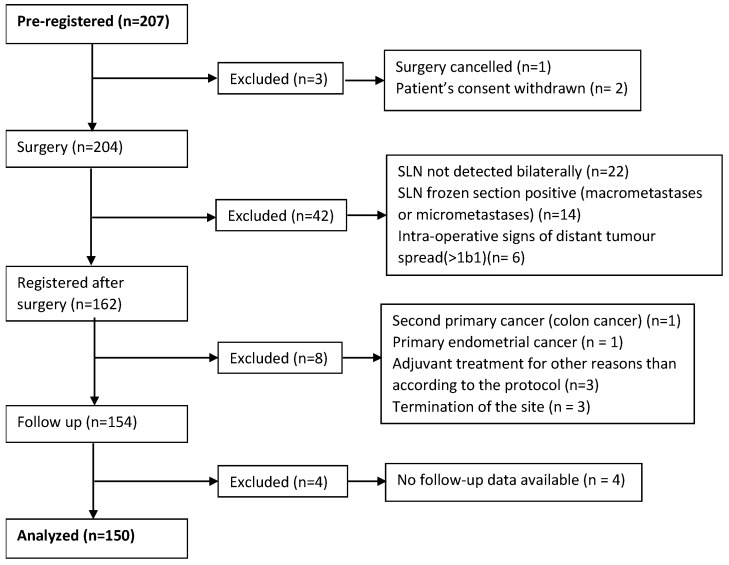
Flow chart of patients registered in the SENTIX trial.

SENTIX (SENTinel lymph node in cervIX cancer) is a prospective, multicenter, observational trial on SLN biopsy in patients with early-stage cervical cancer, with the primary endpoint being the recurrence rate at the 24th month of follow-up. The pre-planned secondary endpoint was the assessment of the prevalence of LLL. Cervical cancer patients with FIGO 2009 [32] stage IA1 with lymphovascular space invasion, IA2, or IB1, who lacked suspicious lymph nodes on preoperative imaging, had a common histological tumor type (squamous, adeno- or adenosquamous carcinoma), and the largest tumor diameter less than 4 cm (less than 2 cm for patients scheduled for a fertility-sparing procedure), were pre-registered into the study. All pre-registered patients underwent radical hysterectomy or a fertility-sparing procedure with sentinel lymph node dissection, without PLND. After the surgery, patients were registered if they met additional intraoperative criteria: bilateral SLN detection, no metastasis of any size found on frozen sections, and no evidence of more advanced disease (Figure 1). All retrieved sentinel lymph nodes were consequently processed by ultrastaging protocol, as described elsewhere [24].

### 2.2. Objective Secondary LLL Assessment

Limb volume assessment was performed at the preoperative visit and subsequently during follow-up visits, every six months for two years after surgery. Each limb was measured at five standardized levels (C1–C5): C1—10 cm below anterior superior iliac spine; C2—10 cm above the midpoint of the knee joint; C3—10 cm below the midpoint of the knee joint; C4—10 cm above the medial ankle (malleolus); C5—level of the medial ankle (malleolus) (Figure S1). All measurements were performed under standardized conditions: (i) a flexible tape measure was used; (ii) the patient was lying still in a supine, relaxed position with the leg straight; (iii) markings were applied on the skin in five defined levels; and (iv) the tape was kept at right angles to the limb length axis in all girth measurements.

The total limb volume was calculated as a sum of the volumes of four individual segments (Equation (1)). The volume of each limb segment was calculated based on the truncated cone formula, where *h* = height of the segment, *A* = circumference at the top of the segment, *B* = circumference at the bottom of the segment.

(1) $$V_{limb}=\sum \frac{\left(h\right)*(A^{2}+A*B+B^{2})}{12*\pi }$$

Above-knee volume (thigh volume) was calculated as a volume of the segment between the C1 and C2 levels. The below-knee volume (calf and ankle volume) was calculated as a sum of segments C3–C4 and C4–C5.

Lymphedema was classified at 24-months follow-up as a persistent limb volume increase (LVI) of 10–19% (mild LLL), 20–39% (moderate LLL), and >40% (severe LLL) between preoperative and postoperative assessments [33,34]. Chosen threshold of 10% LVI was based on a prior paper of Spillane et al. [35] who correlated volume thresholds with patient-reported outcomes >7% increase in the sum of circumferential measurements provided a robust definition of LLL with a sensitivity of 50% and specificity of 100%. >7% increase in the sum of circumferential measurements equals 10–12% of LVI.

Persistent LVI was characterized as a volume change persisting over the period of at least six months, i.e., observed at least during the two consequent follow-up visits.

### 2.3. Patient-Reported LLL

At each follow-up visit, all patients were inquired using an unvalidated questionnaire for their subjective assessment of each lower leg swelling during the past six months, separately for five levels: loin, thigh, calf, ankle, and foot. Should the patient report swelling in either of the levels at least at two follow-up visits, the subjective assessment was considered positive.

### 2.4. Statistics

Standard descriptive statistics were applied in the analysis: absolute and relative frequencies for categorical variables and median supplemented by 5th–95th percentile range for continuous variables. The statistical significance of the relationship between the objective and subjective evaluation of lymphedema was tested using Fisher’s exact test and the Pearson correlation coefficient for binary data and its statistical significance. The predictive power of patient characteristics for lymphedema endpoint occurrence was analyzed using logistic regression and described by odds ratios and their 95% confidence interval and statistical significance. Kaplan–Meier methodology was adopted for the description of time to lymphedema development. α = 0.05 was adopted as a level of statistical significance in all analyses. The analysis was computed using SPSS 25.0.0.1 (IBM Corporation, 2019) and R with package ggplot2.

### 2.5. Study Approval

The protocol was approved by the Institutional Review board of the leading institution (General University Hospital in Prague, Prague, Czech Republic) in 2015. Institutional review board approval of all participating sites was a prerequisite for participation. All participating patients signed informed consent before enrolling in the study. The study was performed in accordance with the Declaration of Helsinki. SENTIX is conducted as a European Network of Gynecological Oncology Trial Groups (ENGOT) trial (ENGOT Cx2) and is led by the Central and Eastern European Gynecologic Oncology Group (CEEGOG; CEEGOG Cx1). The study is performed according to ENGOT Model A [36].

## 3. Results

### 3.1. Patient Characteristics

We analyzed the data from 150 patients treated per protocol and followed for at least 24 months in a prospective international multicentric trial on SLN biopsy in cervical cancer (SENTIX). The characteristics of the patients are summarized in Table 1. The majority of patients had pre-treatment FIGO stage IB1 (128 patients/85.3%), preoperative tumor size ≤2 cm (107/71.3%), and squamous cell tumor histotype (102/68.0%). The median number of removed SLNs per patient was three (5th–95th percentile: 2–6.55). The most common localization of SLN was the interiliac region (46.7%), followed by the external iliac region (42.5%) and the common iliac region (10.2%). Only two of all removed SLNs were localized in the presacral region.

Using ultrastaging protocol [37], nine patients (6%) were diagnosed with SLN metastasis and treated with adjuvant treatment (eight chemoradiotherapy; one combined radiotherapy). In four patients, isolated tumor cells were detected, though no adjuvant treatment was administered. Adjuvant treatment was administered to six additional patients with positive vaginal margins (two chemoradiotherapy, three combined radiotherapy, one brachytherapy) and three with parametrial tumor involvement (two chemoradiotherapy, one combined radiotherapy). Six out of twenty-eight patients after a fertility-sparing procedure conceived during the follow-up period. Fourteen patients experienced recurrence; three of these died of the disease (Table 1).

### 3.2. Objective Assessment of LLL

**Figure 2:**
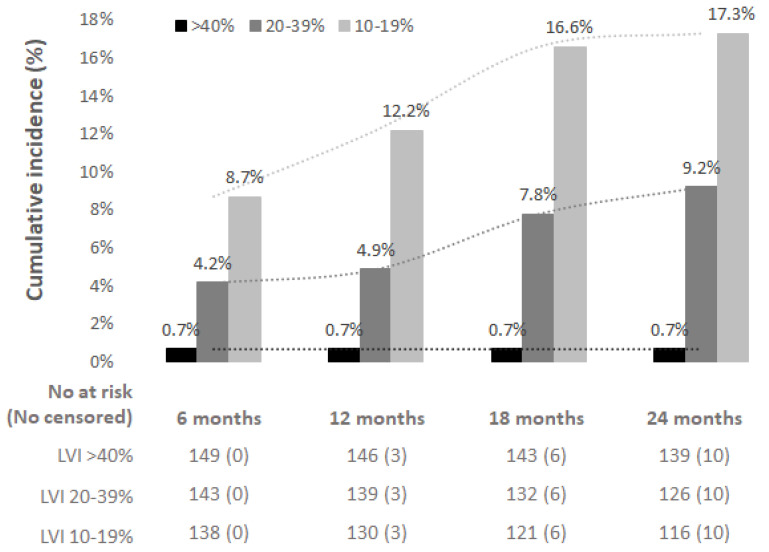
Cumulative incidence of lower-limb lymphedema (LLL) during 24 months of postoperative follow-up. Black: severe LLL (LVI > 40%); dark grey: moderate LLL (LVI 20–39%); light grey: mild LLL (LVI 10–19%). LVI: limb volume increase.

The incidence of LLL, classified according to persistent limb volume increase (LVI) as mild (LVI 10–19%), moderate (LVI 20–39%), or severe (LVI > 40% LLL), is shown in Figure 2. Individual LVI changes in detail are shown in Figure S2. Overall, 24 patients (cumulative incidence 17.3%) experienced persistent mild LLL, 13 patients (9.2%) moderate, and one patient (0.7%) severe LLL. In the moderate and severe LLL groups, 9/14 patients had bilateral LLL and 5/14 had LLL localized unilaterally, all in the right limb. All patients with unilateral moderate LLL experienced mild LLL in the second extremity. In patients with LLL, two received adjuvant radiotherapy. In one case, the onset of LLL coincided with pregnancy, and in one patient, limb swelling coincided with leg thrombosis (Table S1). Three of the LLL patients experienced recurrence during the 24 months of the follow-up; time of the recurrence in neither case corresponded to LLL development.

The median interval to LLL onset was nine months (95% CI: 7.0–11.0): 50% occurred during the first six months, 15.8% between 6–12 months, 26.3% between 12–18 months, and 7.9% between 18–24 months after surgery (Figure 1). We did not report any significant tendency for more frequent or earlier swelling in either pre-defined part of the limb (bellow knee vs. above knee) (Figure S3).

Transient edema resolving without intervention during the six-month period was observed in 22% of patients (either unilaterally or bilaterally) (Figure S4).

### 3.3. Subjective Assessment of LLL

**Figure 3:**
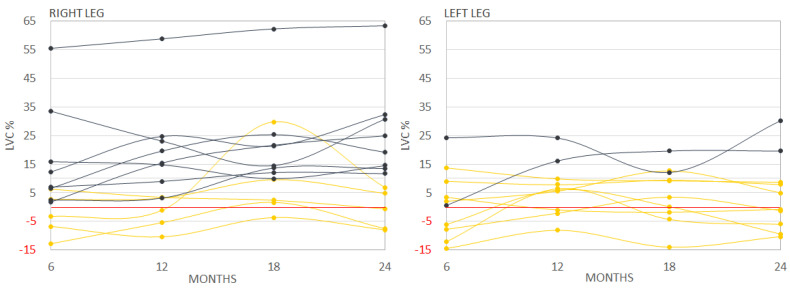
Individual fluctuations of objectively assessed LVC in patients subjectively reporting LLL. Yellow lines: patients without objective LLL; Black lines: patients with objective LLL (all grades). LVC: limb volume change; LVI: limb volume increase.

Only 16 patients (10.7%) subjectively reported LLL; eight patients in both limbs and five and three unilaterally in their right and left limb, respectively. In 50% of patients reporting LLL (8/16), persistent LVI was not objectively observed, frequently reaching negative limb volume change values (Figure 3). Only 16.7% and 23.1% of patients with mild and moderate objectively assessed LLL reported LLL subjectively (Table S1). Interestingly, the only patient with objective severe LLL reported subjective LLL only for the right limb even though the LVI was comparable (LVI > 60%) for both limbs (patient 1, Figure S2).

Only a weak correlation between the subjectively reported and objectively assessed presence of LLL was found, though only for the right limb. This correlation did not remain positive for the mild LLL subgroup, only being observed in moderate and mild + moderate groups (Table 2).

### 3.4. Factors Predisposing to LLL

Logistic regression was employed to ascertain the significance of the inherent prognostic variables (age, BMI, surgical approach, FIGO stage, side-specific number of removed SLNs, radicality of the surgery, adjuvant treatment, tumour size, tumour histotype, grade, LVSI) on the development of objectively assessed LLL. Only an open surgical approach was a marginally significant protective factor for the development of below-knee lymphedema on either limb, with hazard ratio [HR]: 0.255; confidence interval [CI] 0.069, 0.942, *p* = 0.04 (right leg); and HR 0.27, CI 0.073, 1.0, *p* = 0.05 (left leg) (most relevant tested parameters are summarized in Table 3; full data can be found in Table S2). Neither BMI, number of removed SLNs, or administration of adjuvant therapy were significant.

## 4. Discussion

This is the first prospective multicenter study evaluating LLL incidence in cervical cancer patients after a uterine procedure with SLN biopsy, without systematic PLND. The group of 150 patients was prospectively evaluated using both objective and subjective LLL assessments.

The study revealed that LLL develops even after the SLN biopsy only; cumulative incidence at 24-months post-surgery reached 17.3% for mild and 9.2% for moderate LLL, while severe LLL developed in one patient only (0.7%). The median LLL onset time in the whole cohort was nine months. The risk of LLL development was not impacted by other factors such as the number of removed SLNs, surgical approach, adjuvant radiotherapy, type of uterine procedure, age, or BMI. Only 10.7% of patients subjectively reported LLL, and we found a weak correlation between objectively assessed and patient-reported LLL only for the right leg in moderate and mild + moderate subgroups. The important finding of our study was the high rate of transient edema, observed in 22% of the patients and characterized as a single episode of swelling that resolved without intervention until the next follow-up visit.

The incidence of LLL after standard surgical treatment, including PLND, of cervical cancer varies broadly in the literature, in the range of 3–62% [7,8,9,10,11,12,13,14,15,25,26,31]. Such great variation is likely attributed to the flaws in LLL assessment, such as the absence of objective assessment methods, the simple postoperative assessment instead of longitudinal repeated follow-up, as well as a short period between the surgery and endpoint assessment. Moreover, out of more than 30 studies published since 2010 that report LLL incidence in cervical cancer survivors, the majority are based on retrospective cohorts in which the methodology of LLL evaluation is described vaguely or is completely missing [8,26,31,38,39,40,41,42].

Out of ten identified prospective studies, the description of LLL assessment methodology is only vaguely portrayed in two [43,44]; three studies were based on subjective reports [45,46,47]; and only two were based on objective diagnostic methods (bioimpedance spectroscopy, CT, or MRI) [25,48]. The last three prospective studies utilized objective assessment using circumferential measurements [7,10,19]. In the first study, 34.8% (48/138) of patients developed LLL during the two years of follow-up [10]. In this study, however, any exceedance of the >10% threshold at any of seven follow-up visits was considered LLL, which undoubtedly led to an overestimation of the results because transient edema cases were not excluded. In the second study of only 39 patients, the prevalence of LLL was similar (35.9%). The biggest weakness of this study, apart from the limited cohort, is the short follow-up of only six months, which could again lead to LLL prevalence overestimation since the swelling of the limbs in the first year post-surgery is very likely to be transient [49]. In the last study, LLL one-year post-surgery was identified in 34.5% of patients (20/58). However, in this study, a low threshold of >2% LVI was adopted in the more affected limb accompanied by other LLL symptoms [7].

Data on the risk of LLL in cervical cancer patients after SLN biopsy without simultaneous PLND are still scarce, yet they all conclude on a positive impact of decreased radicality of lymph node staging on the risk of LLL [25,26,31,50]. Again, however, all previously published studies have serious methodological limitations.

In a retrospective Japanese study, none out of 70 patients after SLN biopsy developed LLL as compared to 13.4% of 97 patients after systematic PLND [26]. A similar outcome was reported in another retrospective cohort, reporting zero (0/139) occurrence versus 22.4% (15/67) of LLL patients after SLN biopsy and PLND, respectively [31]. In the first paper, the LLL assessment methodology description is limited to the statement that lymphatic complications were assessed using International Society Lymphology Guidelines [2]. In the second study, the method used for LLL detection is completely missing.

The risk of LLL development after SLN biopsy was also studied in two prospective studies. In the first cohort of 35 patients, new symptomatic LLL (stage II and above) was identified at 42 months in two of the 23 (8.7%) cervical cancer patients after SLN biopsy, but in five of 12 patients (42%) after PLND [25]. However, the groups were not well balanced in terms of risk factors, and the PLND group received adjuvant treatment significantly more often [25]. The second study, SENTICOL II, comparing outcomes of 101 patients after PLND and 106 patients after SLN biopsy, concluded that LLL symptoms reported by patients were significantly less severe after SLN biopsy. This outcome was based on the evaluation of a quality-of-life questionnaire, while the differences in objectively measured thigh circumferences were not significant [50]. Nevertheless, the objective assessment of LLL was based on only two circumferential measurements of the thigh, not allowing for limb volume calculation. Assessments were performed only in a limited period of 6 months after the surgery, and, finally, preoperative measurements were compared to a maximal value acquired at any of the three measurements performed early after surgery (at 1, 3, 6 months), therefore not excluding the patients with transient edema [50].

A substantially favorable impact of SLN biopsy on LLL risk was also reported for other pelvic gynecologic cancers [51]. A prospective study of 188 endometrial cancer patients revealed that replacement of PLND by SLN biopsy decreased LLL incidence up to 14-fold [28]. However, the compared cohorts were not equal in prognostic factors, which, among other things, caused more frequent administration of adjuvant radiotherapy in the PLND group (58.7% vs 13.9%) [28]. In another large cohort of 2535 vulvar cancer patients, a five-fold lower risk of LLL was observed in the SLN group compared to patients after inguinofemoral lymph node dissection [27]. In this meta-analysis, studies with a wide variety of objective diagnostic and rating criteria were combined; hence, at least some bias in the comparisons is expected [27].

Overall, preliminary data from retrospective and small prospective cohorts in gynecological cancer raised expectations for the elimination of the risk of LLL after SLN biopsy. This anticipation was, unfortunately, not confirmed by our study. Although a severe form of persistent LLL was almost not present (a single case, 0.7%), a mild or moderate form occurred in a total of 26.5% of cases. Nevertheless, we are not the first study reporting a substantial risk of LLL after SLN biopsy in the pelvis. A similar incidence (25% vs. 24%) of LLL was observed in a prospective study on 97 endometrial cancer patients after both techniques of lymph node staging (i.e., SLN biopsy and PLND) [52].

Our study also revealed that patient-reported and objective LLL have to be clearly distinguished. Cumulative incidence at 24 months was 27.2% for objective LLL while it was 10.7% for self-reported LLL, showing only a weak and partial correlation (observed only for right leg) between those two types of assessment modalities. Only 50% of patients self-reporting LLL had LLL confirmed objectively. Out of patients with moderate/severe LLL and mild LLL, only 21.7% and 19.0% subjectively reported edema.

The relationship between objective limb volume changes and subjective symptoms was previously studied in a prospective study of 136 cervical cancer patients utilizing the Gynecologic Cancer Lymphedema Questionnaire (GCLQ), which consists of numerous LLL-related symptoms [53,54]. As much as 48% of all patients had a GCLQ score increment ≥4 points during a postoperative assessment, but less than half of them had LVI 10%. On the other hand, 60% of LVI > 10% of patients reported LLL on the GCLQ. The prospective EMBRACE study of locally advanced cervical cancer reported that prevalence of LLL at five years after the end of treatment was 15% when assessed by a physician and 34% using patient-reported symptoms (EORTC QLQ-CX24) [55]. Four percent of patients were not diagnosed with LLL; however, they reported quite a bit/very much swelling of the limb. At the same time, 57.7% of mild LLL and 22.4% of moderate LLL patients reported no/only a little limb swelling [55]. Other studies assessing both objective ad subjective LLL in cervical cancer survivors do not allow for correlation analyses between them, not providing the information about the number of patients with either or both positive assessments [48,56].

Numerous risk factors related to LLL were previously described in the literature, such as adjuvant radiotherapy [10,57], the extent of PLND with the emphasis on the number of removed LN [9,15,20,21,22,23], removal of circumflex iliac nodes [9,16,41], FIGO stage [23], and increasing BMI [12,57]. In our study, none of the tested factors was proven significant. The only exception was the surgical approach, where laparotomy was inversely related to below-knee edema. This was, however, only marginally significant and lacks justification why should minimally invasive surgery be a risk factor for LLL development. The lack of association between radiation therapy and LLL development was likely due to the low rate of adjuvant treatment administration in the whole study cohort (only 12% of patients). Most importantly, the number of removed SLNs on a respective side did not correlate with LLL development in the limb. Therefore, we can conclude that number of removed SLNs (1.5 per side on average; 3 per patient) is already below the risk number threshold, which was previously described in the literature to be between 10 to 31 [9,15,20,21,22,23].

Our study represents the biggest reported prospective cohort of cervical cancer patients treated by uterine surgery with SLN biopsy only, who were prospectively evaluated by standardized objective and subjective LLL assessment methods. In order to overcome the previously mentioned limitations in the methodology, we employed a highly sensitive LLL assessment method based on serial circumferential measurements of the limbs, measured in six-month periods over the course of 24 months. The used threshold of 10% LVI is based on a prior correlation of volume thresholds with patient-reported outcomes, which showed that an increase of >7% in the circumferential sums provides a clinically meaningful definition of LLL (converted, >7% circumferential increase approximately corresponds to LVI of >10–12%) [35]. Frequent, repeated assessments also allowed us to distinguish LLL from transient edema from other causes. Another important advantage is a precise standardization of this objective assessment method. Circumferential measurements in other studies were done in 10 cm intervals, usually starting from the medial ankle, and therefore not measuring comparable anatomical regions across patients with different leg lengths. We adopted this technique and measured circumferences at five anatomically standardized levels based on bony landmarks, allowing for unbiased inter- and intra- individual comparisons based on the comparable anatomical sections.

Amongst limitations, due to the multicenter design, circumference measurements were taken by dozens of investigators, which, despite detailed instructions, can lead to inaccuracies in individual measurements. The questionnaire used for the assessment of subjective patient-reported LLL was not standardized, and patients reported only the presence of swelling at different levels corresponding to sections used for objective measurement. Finally, due to the observational character, our study does not allow for comparison with the control group of patients after systematic pelvic lymphadenectomy. Therefore, the results can only be compared with previously published data, which are mainly retrospective and varying in diagnostic and assessment methods, meaning that direct comparison of the results is imprecise.

## 5. Conclusions

In conclusion, our study showed that PLND replacement by SLN biopsy in the surgical treatment of cervical cancer does not eliminate the risk of LLL development. Cumulative incidence of mild and moderate LLL at 24 months reached 17.3% and 9.2%, respectively, with a median onset time of nine months after surgery.

The high rate of transient edema and the weak correspondence of patient-reported symptoms with the objective findings emphasize that a reliable LLL assessment requires a standardized methodology based on objective and repeated measurements.

## Figures and Tables

**Table 1:** Characteristics of patients (*n* = 150).

Parameter	Category	*n* (%)
Age category (years)	≤40	54 (36.0%)
	41–60	78 (52.0%)
	>60	18 (12.0%)
Body mass index category (kg/mg^2^)	≤25	79 (52.7%)
	26–30	41 (27.3%)
	>30	30 (20.0%)
ECOG performance status	0	146 (97.3%)
	1	4 (2.7%)
FIGO stage (preoperative)	IA1 + LVSI	10 (6.7%)
	IA2	12 (8.0%)
	IB1	128 (85.3%)
Grade	G1	32 (21.3%)
	G2	85 (56.7%)
	G3	33 (22.0%)
Tumor type	Squamous cell carcinoma	102 (68.0%)
	Adenocarcinoma	46 (30.7%)
	Adenosquamous carcinoma	2 (1.3%)
Tumor size (preoperative imaging)	≤2 cm	107 (71.3%)
	>2 cm	43 (28.7%)
Lymphovascular space invasion (LVSI)	Yes	40 (26.7%)
	No	110 (73.3%)
Surgical approach	Laparotomy	50 (33.3%)
	Minimally invasive	100 (66.7%)
Type of uterine procedure	Type B radical hysterectomy	36 (24.0%)
	Type C1 radical hysterectomy	61 (40.7%)
	Type C2 radical hysterectomy	24 (16.0%)
	Simple hysterectomy	1 (0.7%)
	FST (conization, trachelectomy)	28 (18.6%)
No of removed SLNs	2	59 (39.3%)
	3–4	69 (46.0%)
	>4	22 (14.9%)
SLN metastatic involvement	No	137 (91.3%)
	Macrometastasis or micrometastasis	9 (6.0%)
	Isolated tumor cells	4 (2.7%)
Adjuvant treatment	Chemoradiotherapy	12 (8.0%)
	Combined radiotherapy	5 (3.3%)
	Brachytherapy	1 (0.7%)
	None	132 (88.0%)
Pregnancy	No	144 (96.0%)
	Yes	6 (4.0%)
Recurrence	No	136 (90.7%)
	Yes	14 (9.3%)
Death of disease	No	147 (98.0%)
	Yes	3 (2.0%)

BMI: body mass index; ECOG: Eastern Cooperative Oncology Group; FST: fertility-sparing treatment; LVSI: lymphovascular space invasion; SLN: sentinel lymph node.

**Table 2:** Correlation between subjective and objective LLL assessment.

Right Limb								
		Subjective		Total	% Sub.	*p*-Value (Fisher Test)	Pearson Correlation	*p*-Value
		No	Yes					
LVI > 20%	no	127	9	136	6.6%			
	yes	10	4	14	28.6%	0.021	0.227	0.005
LVI 10–19%	no	120	9	129	7.0%			
	yes	17	4	21	19.0%	0.087	0.149	0.069
LVI > 10%	no	110	5	115	4.3%			
	yes	27	8	35	22.9%	0.002	0.278	0.001
**Left Limb**								
		**Subjective**		**Total**	**% Sub.**	***p*-Value (Fisher Test)**	**Pearson** **Correlation**	***p*-Value**
		**No**	**Yes**					
LVI > 20%	no	131	10	141	7.1%			
	yes	8	1	9	11.1%	0.506	0.037	0.656
LVI 10–19%	no	120	9	129	7.0%			
	yes	19	2	21	9.5%	0.653	0.034	0.680
LVI > 10%	no	112	8	120	6.7%			
	yes	27	3	30	10.0%	0.460	0.051	0.534

LVI: limb volume increase; sub.: subjective; subjective: subjective assessment. Statistical significance level *p* ≤ 0.05.

**Table 3:** Risk factors analysis associated with objective LLL at 24 months follow-up.

	Age Categories (Reference ≤ 40)		BMI Categories (Reference ≤ 25)		Surgical Approach (Reference MIS)		FIGO Stage (Reference IA2 + IA1 + LVSI)	No SLN (Reference ≤ 2)	Radical Parametrectomy (Reference B)			Adjuvant Radiotherapy (Reference No)
	41–60	>60	26–30	>30	Laparotomy		IB2	>2	C1	C2	Not done	Yes
LVI Localization	*p*	*p*	*p*	*p*	*p*	OR (95% CI)	*p*	*p*	*p*	*p*	*p*	*p*
Right above knee	0.129	0.496	0.754	0.299	0.786	0.854 (0.274; 2.666)	0.898	0.213	0.637	0.486	0.165	0.473
Right below knee	0.011	0.975	0.813	0.389	0.040	0.255 (0.069; 0.942)	0.368	0.233	0.893	0.416	0.073	0.774
Left leg above knee	0.382	0.402	0.710	0.590	0.273	1.968 (0.586; 6.608)	0.585	0.789	0.531	0.894	0.721	0.644
Left below knee	0.086	0.771	0.237	0.335	0.050	0.270 (0.073; 1.000)	0.237	0.968	0.065	0.168	0.732	0.571
Right leg sum	0.050	0.224	0.510	0.261	0.956	0.968 (0.304; 3.080)	0.988	0.580	0.292	0.447	0.053	0.546
Left leg sum	0.112	0.942	0.618	0.977	0.502	1.540 (0.437; 5.431)	0.697	0.463	0.655	0.337	0.288	0.728

Statistical significance level *p* ≤ 0.05.

## Data Availability

The data sets used and/or analyzed during the current study are available from the corresponding author on reasonable request.

## Supplementary Materials

The following are available online at https://www.mdpi.com/article/10.3390/cancers13102360/s1, Figure S1: Secondary lymphedema assessment schema, Figure S2: Individual fluctuations of limb volume change in patients with objective LLL, Figure S3: Distribution of the 10–19% and >20% volume increase of right and left leg divided into above- and below- knee segments, Figure S4: Incidence of transient edema, Table S1: Objective LLL onset stratified by the laterality and overview of predisposing risk factors, Table S2: Risk factors analysis associated with objective LLL at 24 months follow up—full data.
