# Supplementary material for: Lower-Limb Lymphedema after Sentinel Lymph Node Biopsy in Cervical Cancer Patients
Source: Cancers (Basel). 2021 May 13;13(10):2360. doi: 10.3390/cancers13102360 (PMC8153612; doi:10.3390/cancers13102360)
Supplement: Supplementary file 1 [file cancers-13-02360-s001.zip › cancers-1193287-supplementary.pdf]

Supplementary material

Title: Lower-limb lymphedema after sentinel lymph node biopsy in cervical cancer patients

David Cibula, Martina Borčinová, Simone Marnitz, Jiří Jarkovský, Jaroslav Klát, Radovan Pilka, Aureli Torné, Ignacio Zapardiel, Almerinda Petiz, Laura Lay, Borek Sehnal, Jordi Ponce, Michal Felsinger, Octavio Arencibia Sánchez, Peter Kaščák, Kamil Zalewski, Jiri Presl, Alicia Palop Moscardó, Solveig Tingulstad, Ignace Vergote, Mikuláš Redecha, Filip Frühauf, Christhardt Köhler, and Roman Kocián

Correspondence: David Cibula, Department of Obstetrics and Gynecology, First Faculty of Medicine, Charles University and General University Hospital, Apolinarska 18, Prague 2, 12801, Czech Republic. Tel.: +420224967451. email: dc@davidcibula.cz

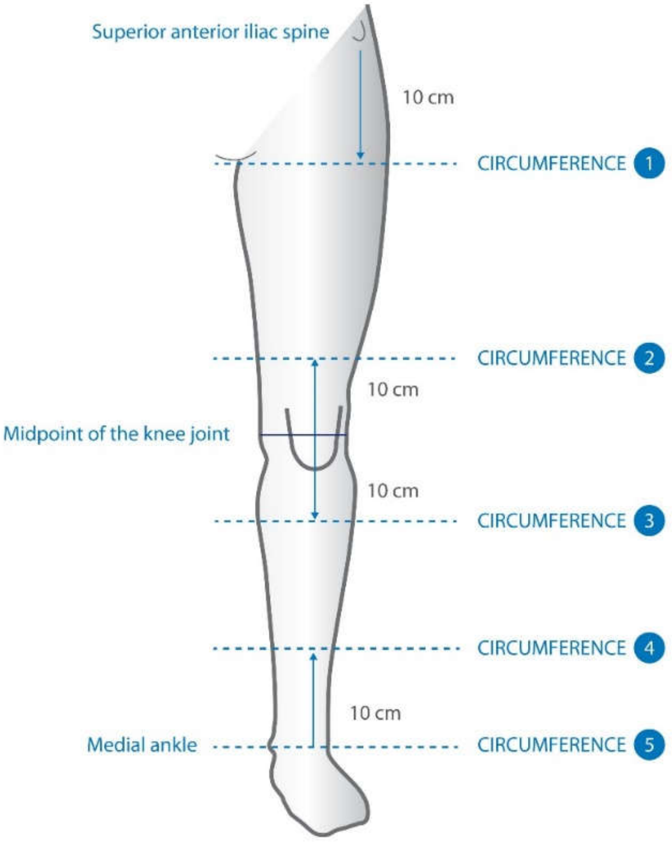

Figure S1. Secondary lymphedema assessment schema

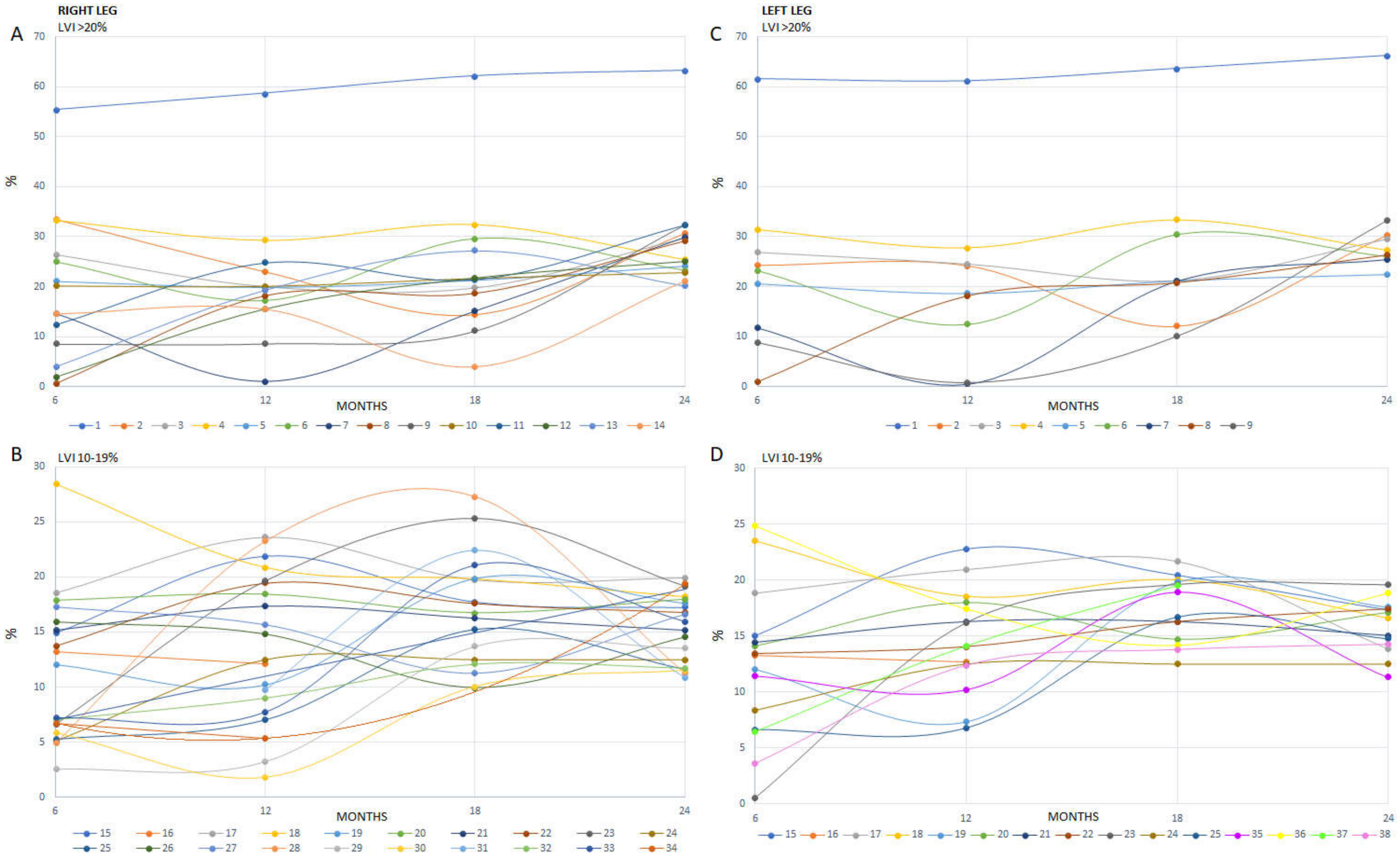

Figure S2. Individual fluctuations of limb volume change in patients with objective LLL. **A**: right leg LVI >20%; **B**: right leg LVI 10-19%; **C**: left leg LVI >20%; **D**: left leg LVI 10-19%. Each number represents unique a patient identification number and matches patient numbering in Supplementary Table 1.

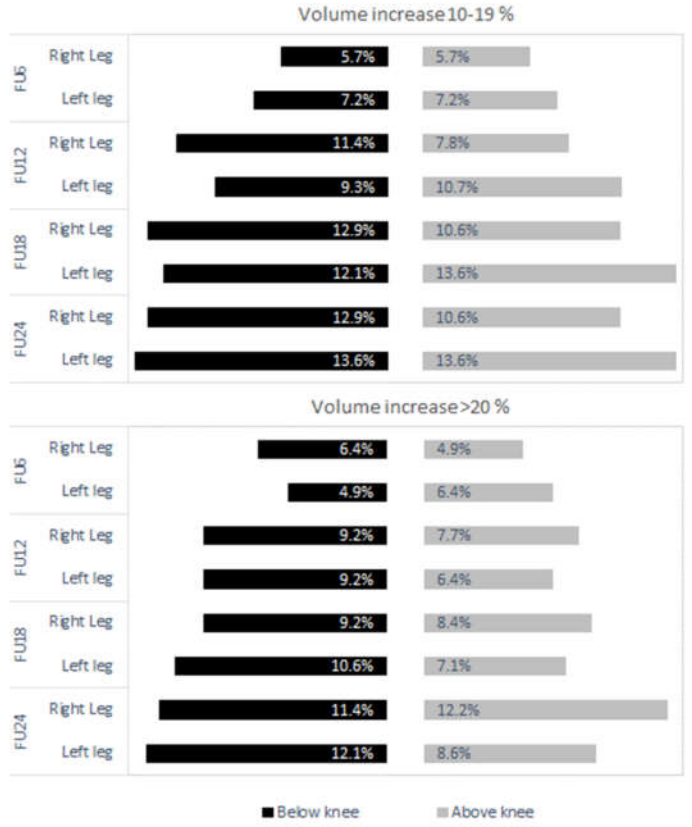

Figure S3. Distribution of the 10–19% and >20% volume increase of right and left leg divided into above- and below- knee segments.

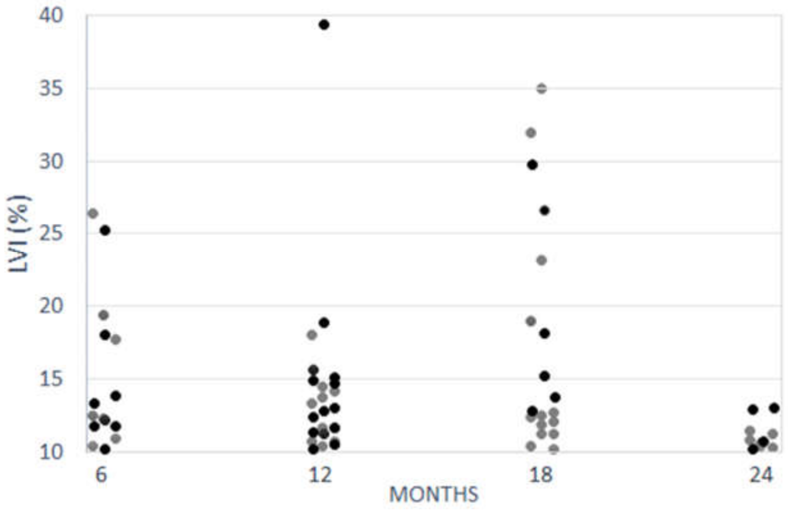

Figure S4. Incidence of transient edema. Each dot marks the transient oedema (>10% limb volume increase) in an individual patient resolved without intervention within the period between two consequent follow-up measurements. Grey dots: left leg; black dots: right leg.

Table S1. Objective LLL onset stratified by the laterality and overview of predisposing risk factors. White fill marks LVI below 10%; light grey 10-19%; dark grey 20-39%; black >40%.

Patient	Right Leg objective						Left Leg objective						
no.	cluster	FU 6	FU12	FU18	FU24	subj	cluster	FU 6	FU12	FU18	FU24	subj	Risk factors
1	>40%					pos	>40%					neg	adjuvant combined RT
2	20-39%					pos	20-39%					pos	recurrence FU18
3	20-39%					neg	20-39%					neg	none
4	20-39%					neg	20-39%					neg	none
5	20-39%					neg	20-39%					neg	none
6	20-39%					neg	20-39%					neg	none
7	20-39%					neg	20-39%					neg	none
8	20-39%					neg	20-39%					neg	none
9	20-39%					neg	20-39%					neg	none
10	20-39%					neg	10-20%					neg	none
11	20-39%					pos	10-20%					neg	none
12	20-39%					pos	10-20%					pos	none
13	20-39%					neg	10-20%					neg	none
14	20-39%					neg	10-20%					neg	none
15	10-19%					neg	10-19%					neg	none
16	10-19%					neg	10-19%					neg	recurrence FU12
17	10-19%					neg	10-19%					neg	none
18	10-19%					neg	10-19%					neg	none
19	10-19%					neg	10-19%					neg	none
20	10-19%					neg	10-19%					neg	none
21	10-19%					neg	10-19%					neg	none
22	10-19%					neg	10-19%					neg	none
23	10-19%					pos	10-19%					pos	none
24	10-19%					neg	10-19%					neg	none
25	10-19%					neg	10-19%					neg	none
26	10-19%					pos	none					pos	none
27	10-19%					neg	none					neg	none
28	10-19%					neg	none					neg	none
29	10-19%					pos	none					neg	adjuvant combined RT
30	10-19%					neg	none					neg	recurrence FU24
31	10-19%					neg	none					neg	none
32	10-19%					pos	none					pos	pregnancy FU18-FU24
33	10-19%					neg	none					neg	none
34	10-19%					neg	none					neg	none
35	none					neg	10-19%					neg	none
36	none					neg	10-19%					neg	none
37	none					neg	10-19%					neg	thrombosis FU12-FU18
38	none					neg	10-19%					neg	none

FU6: follow up at 6 months post-surgery; FU12: 12 months; FU18: 18 months; FU24: 24 months; LVI: persistent limb volume increase; pos: positive; neg: negative; RT: radiotherapy; subj: subjective LLL assessment.

Table S2. Risk factors analysis associated with objective LLL at 24 months follow up – full data.

	Age categories (reference ≤40)		BMI categories (reference ≤25)		Surgical approach (reference MIS)		FIGO stage (reference IA2 + IA1 + LVSI)	No SLN (reference ≤2)	Radical parametrectomy (reference B)			Adjuvant radiotherapy (reference No)	Tumour size (reference < 2 cm)	Tumour type (reference Squamous cell carcinoma)	Grade (reference G1)		LVSI (reference No)
	**41–60**	**>60**	**26–30**	**>30**	**Laparotomy**		**IB2**	**>2**	**C1**	**C2**	**Not done**	**Yes**	**≥ 2 cm**	**Adenocarcinoma usual type**	**G2**	**G3**	**Yes**
**LVI localization**	***P***	***P***	***P***	***P***	***P***	OR (95% CI)	***P***	***P***	***P***	***P***	***P***	***P***	***P***	***P***	***P***	***P***	***P***
Right above knee	0.129	0.496	0.754	0.299	0.786	0.854 (0.274; 2.666)	0.898	0.213	0.637	0.486	0,165	0.473	0.807	0.690	0.354	0.354	0.756
Right below knee	0.011	0.975	0.813	0.389	0.040	0.255 (0.069; 0.942)	0.368	0.233	0.893	0.416	0.073	0.774	0.264	0.252	0.315	0.669	0.662
Left leg above knee	0.382	0.402	0.710	0.590	0.273	1.968 (0.586; 6.608)	0.585	0.789	0.531	0.894	0.721	0.644	0.908	0.695	0.352	0.722	0.513
Left below knee	0.086	0.771	0.237	0.335	0.050	0.270 (0.073; 1.000)	0.237	0.968	0.065	0.168	0.732	0.571	0.807	0.690	0.714	0.722	0.756
Right leg sum	0.050	0.224	0.510	0.261	0.956	0.968 (0.304; 3.080)	0.988	0.580	0.292	0.447	0.053	0.546	0.449	0.694	0.720	0.261	0.866
Left leg sum	0.112	0.942	0.618	0.977	0.502	1.540 (0.437; 5.431)	0.697	0.463	0.655	0.337	0.288	0.728	0.682	0.378	0.737	0.669	0.756

Statistical significance level $P \leq 0.05$.
